# A journal dedicated to studying the combined effects of activity, sedentary and sleep behaviours

**DOI:** 10.1186/s44167-022-00008-y

**Published:** 2022-09-01

**Authors:** Corneel Vandelanotte

**Affiliations:** Central Queensland University, Appleton Institute, Rockhampton, Australia

The importance of physical activity for health and society has long been recognised [16]. Large amounts of physical activity research have been conducted across all stages of the Behavioural Epidemiology Framework [11], and have been published in public health, psychology, epidemiology and medical journals, as well as journals specifically dedicated to physical activity research. While a landmark study on sedentary behaviour was published as far back as 1953 [6], it wasn’t until much later that it was recognised that physical inactivity and sedentary behaviour weren’t different words for the same thing, but rather that physical activity (or lack thereof) and sedentary behaviour are two distinct, but co-dependent, health behaviours leading to distinct health outcomes [2, 8, 12, 14]. Research on sedentary behaviour started to expand greatly from the early 2000s and in the last two decades there has been a greater than tenfold increase in the number of published papers on ‘sedentary behaviour’ [1]. A recent scoping review identified as much as 108 systematic reviews focussing sedentary behaviour and spanning nearly all the stages of the Behavioural Epidemiology Framework [5]. Yet, there are no journals dedicated specifically to publishing research on sedentary behaviour. Research on sedentary behaviour is published in the same journals where research on physical activity is being published.

More recently researchers recognised that not only are physical activity and sedentary behaviours distinct yet co-dependent behaviours, they are also co-dependent on and interrelated with sleep behaviour [9]. Hence, if one wants to truly understand activity, sedentary and sleep behaviours in terms of health impact, what their combined correlates and determinants are, as well as how to design effective interventions aiming to improve these behaviours simultaneously, they should be studied together, preferably using a 24-h paradigm as they compete for time with one another. Therefore, it is no surprise that the number of research publications focussing on both physical activity and sleep has increased exponentially since 2010 [4]. Moreover, the importance of balancing healthy amounts of physical activity, sedentary behaviour and sleep is increasingly recognised in health guidelines, such as for example guidelines developed by the World Health Organisation, Canada and Australia [7, 10, 13, 17]. Yet, there is no journal that focusses on studying these behaviours in combination.

Therefore, as founding Editor-in-Chief, I’m very proud to be launching the *Journal of Activity, Sedentary and Sleep Behaviors* (JASSB) hosted by BMC, part of Springer Nature. A new journal that will not only be open to accept research publications focussing on physical activity, but also sedentary behaviour and sleep, and with a higher priority offered to studies examining two or three of these behaviours in combination. The journal will especially embrace the development of knowledge and research that adopts a 24-h approach when examining or manipulating physical activity, sedentary and sleep behaviours and their effect on health. JASSB welcomes research applying a broad range of study designs, including randomised controlled trials, epidemiological studies, cross-sectional surveys, qualitative approaches, systematic reviews and compositional data analysis, as well as studies focussing on assessment technology or interventions with a focus on digital health. JASSB accepts original research manuscripts, short reports, reviews, commentaries and study protocols. Moreover, JASSB is a fully open access journal ensuring that the articles are freely available to researchers and readers from all over the world and all articles submitted to the journal will undergo a rigorous peer review.

While the development of any new journal takes time, I’m nevertheless pleased with the nature and quality of the submissions we’re already receiving for the journal. I’m also pleased that some of the first research papers JASSB will publish focus on 24-h movement behaviours. For example, Kuzik et al. [3] investigated how adherence to 24-h movement guidelines is associated with physical, cognitive and social development indicators in 3-to-5-year old’s and found that meeting both sleep and physical activity recommendations was positively associated with physical and overall development. Another example by Tyler et al. [15] investigated associations between 24-h activity compositions and motor competence in children and adolescents and found that reallocations of low intensity physical activity or sleep to moderate-to-vigorous physical activity were associated with the largest increases in motor competence.

There is no doubt that research examining the combined effects of activity, sedentary and sleep behaviours will continue to grow rapidly in the future. Therefore, with a strong international Editorial Board (https://jassb.biomedcentral.com/about/editorial-board), with a focus on quality over quantity, and with an aim to be indexed in all major databases as soon as possible, I’m confident that JASSB will quickly grow and go from strength to strength over the years to come. I hope you can join me in building a strong high-quality journal in this area, and I look forward to seeing your research published in JASSB.
